# Double effects of O_2_ on passive film of super 13Cr stainless steel in CO_2_ saturated environment

**DOI:** 10.1038/s41598-025-01208-7

**Published:** 2025-06-06

**Authors:** Lv Naixin, Fu Anqing, Yu Haitao, Zhang Gang, Wei Chenghua, Wang Xia, Xu Zhengyi, Wang Yanqiu

**Affiliations:** 1 State Key Laboratory of Oil and Gas Equipment, CNPC Tubular Goods Research Institute, Xi’an, 710077 China; 2 Longdong Oil and Gas Development company of Changqing Oil field, Qingyang, 745000 China; 3 Xi’an Changqing Engineering Construction Supervision Co.,Ltd, Xi’an, 710016 China; 4 Associated Gas Comprehensive Utilization Department of Changqing Oil field, Xi ’an, 710016 China; 5 International Joint Laboratory of Advanced Bulk Nanomaterials for Innovative Applications, Harbin Engineering University, Harbin, 150001 China; 6 CEPREI, Guangzhou, 511370 China; 7 Guangzhou Provincial Key Laboratory of Electronic Information Products Reliability Technology, Guangzhou, 511370 China

**Keywords:** Super 13Cr stainless steel, O_2_, Mott-Schottky, Passivation, Pitting, Engineering, Chemical engineering

## Abstract

Herein, a comprehensive suite of electrochemical methods, including cyclic polarization, Mott-Schottky (M-S), and electrochemical impedance spectroscopy (EIS) tests, were employed to investigate the effects of O_2_ on passivation and pitting behavior of Super 13Cr in CO_2_-saturated environment. Our findings reveal that O_2_ plays a dual role: O_2_ substantially enhances the stability of passive film by diminishing point defeat density, thereby improving the resistance to local corrosive attacks. Conversely, within the stable pitting domain, O_2_ exacerbates the severity of the corrosive environment, undermining the repassivation capabilities of super 13Cr stainless steel. The study elucidates the intricate interplay of O_2_ and the passive film, offering valuable insights for the strategic application of Super 13Cr in the demanding conditions of modern oil and gas extraction processes where CO_2_ and O_2_ coexist.

**Supplementary Information:**

The online version contains supplementary material available at 10.1038/s41598-025-01208-7.

## Introduction

The insatiable global demand for energy has spurred the development of advanced oil and gas recovery techniques aimed at enhancing extraction efficiency^1,2^. However, these advanced exploration and production methods have also introduced significant corrosion challenges to downhole tubing and surface gathering pipeline systems, primarily due to the harsh environmental conditions encountered^3^. Stainless steels, known for their excellent mechanical properties, cost-effectiveness, and strong resistance to CO_2_-induced corrosion, have become the preferred materials for these critical applications^4^. Extensive researches have investigated the corrosion behavior of stainless steels under various environmental conditions^5–7^, revealing that the uniform corrosion rate of stainless steels increases with rising temperatures and CO₂ partial pressure^8,9^. Particularly under extreme service conditions, such as 180 °C and 4.16 MPa *P*_CO2_, the corrosion rate significantly increases, and the incidence of pitting corrosion becomes more pronounced^10^.

The susceptibility of metals to corrosion is inherently governed by the characteristics of protective oxide layers that develop on surfaces, especially in highly aggressive environments^11–13^. The properties of corrosion product layers, i.e., composition and structure, are significantly influenced by environmental factors^14,15^. With the advent of polythermal fluid injection and aerated methods to enhance reservoir productivity, oxygen (O_2_) has been introduced into downhole conditions, shifting the traditional corrosion condition from a CO_2_-dominated environment to a mixed O_2_/CO_2_ atmosphere^16–18^. The transition, coupled with the increasing depths of drilling operations and the consequent rise in downhole temperature and pressure, has further complicated the service environment for tubing and exacerbated the severity of service conditions. Field observations have reported incidents of corrosion-induced perforation and catastrophic tubing failures within the O_2_/CO_2_ coexistence environment^19,20^. Li et al.^21^ documented that impurities can mitigate corrosion rates and nodule proliferation, thereby enhancing the stability of chromium oxides. However, Bouhieda et al.^22^ argued that these impurities could accelerate the early development of a protective chromium-rich oxide layer. The finding contrasts with the work of Mahaffey et al.^23^, who identified that the same levels of oxygen impurities can increase the likelihood of nodular defects and internal oxidation, as well as the formation of voids in the matrix, attributed to the accelerated outward diffusion of chromium. These conflicting results highlight the complex role of impurities in the formation and stability of chromium oxides, which is a key consideration in the context of the work.

The study addresses the passivation and pitting mechanism gap of Super 13Cr under 50% N_2_/O_2_ + 50% CO_2_ environment by employing a comprehensive array of electrochemical assays and artificial pitting electrode experiment. The study aims to elucidate the significant effects of oxygen on passive film, utilizing the Point Defect Model (PDM)^24,25^ and Galvele’s local acidification theory^26^ as theoretical frameworks. Our findings underscore the pivotal role of O_2_ in enhancing the passive film stability and intensifying corrosiveness of local environment within pits, providing a theoretical foundation for ensuring the reliable service life of oil pipeline materials in the challenging environments encountered in unconventional oil and gas extraction operations.

## Experimental

### Materials

Super 13Cr martensitic stainless steel (chemical composition detailed in Table 1) was utilized. Electrochemical test specimens were prepared as squares (10 × 10 × 3 mm), sealed, and an area of 10 × 10 mm was exposed for testing. Additionally, an artificial pitting electrode was fabricated from a small round rod with a diameter of 350 μm, resulting in an active area of 9.6 mm^2^. A 0.1 M NaCl solution, prepared using 99.9% pure reagents and deionized water, was used for electrochemical testing. The experimental gas mixtures consisted of 50% O_2_ + 50% CO_2_ and 50% N_2_ + 50% CO_2_. The dissolved oxygen levels in the solutions equilibrated with gas mixtures were 0.3 and 12.6 mg/L, measured using a HACH dissolved oxygen electrode (LDO10101). Both saturated solutions exhibited a pH of 4.2, and the measurements were conducted at 25 ℃. Before electrochemical experiments, deoxygenation process was employed by purging with the mixture gas for 2 h.

**Table 1:** Chemical composition of experimental super 13Cr (wt%).

Element	C	Ni	Cr	Si	Mo	*P*	S	Mn	Fe
Content	0.027	5.32	12.87	0.18	2.20	0.022	0.004	0.47	Bal.

### Electrochemical measurements

Electrochemical experiments were conducted using the P4000 A potentiostat from Princeton Applied Research, with a standard three electrode setup (counter electrode: platinum sheet, reference electrode: solid Ag/AgCl electrode (−0.221 V, in our previous work^27^). Specimens were carefully prepared using SiC sandpaper up to 1500 grit, followed by thorough rinsing and decontamination. To remove the native surface oxide film, the specimens were polarized at −1 V vs. open circuit potential (OCP) for 5 min. The system was then allowed to stabilize for 1 h before tests. Cyclic potentiodynamic polarization tests commenced at −0.15 V vs. OCP with the scan rate of 0.333 mV⋅s^−1^. Reverse scan was initiated once the current density exceeded 1 × 10^−3^ A·cm^−2^. For potentiostatic polarization, samples were held at constant film-forming potentials (*E*_f_) of −0.16, −0.08, 0 and 0.08 V for 2 h to generate polarization curves. Subsequent to polarization, measurements of EIS were performed using a 10-mV amplitude across a frequency spectrum extending from 10^−2^ to 10^5^ Hz. Directly following the potentiostatic polarization, a Mott-Schottky (M-S) test was executed. The test started from the polarization potential and descended to −0.5 V in steps of −0.02 V.

## Results

### Cyclic potentiodynamic polarization curves

**Fig. 1:**
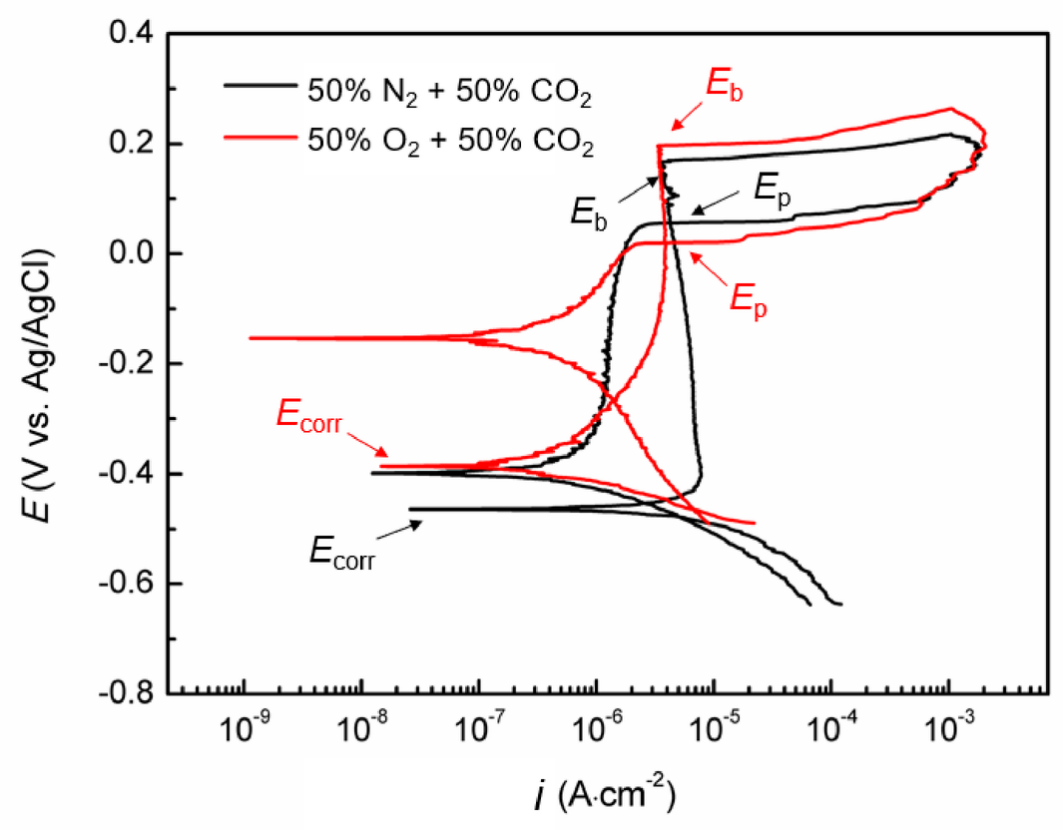
Cyclic potentiodynamic polarization results of Super 13Cr under a 50% N_2_/O_2_ and 50% CO_2_ environment.

Figure 1 illustrates the cyclic potentiodynamic polarization results for Super 13Cr in 0.1 M NaCl solution under the two environments: 50% O_2_ + 50% CO_2_ and 50% N_2_ + 50% CO_2_, which has explained in our previous study^28^. The parameters are detailed in Table 2. In both environments, Super 13Cr stainless steel demonstrates passivation behavior during anodic polarization. The corrosion potential (*E*_corr_) is indicative of the passive film stability^29^. Higher *E*_corr_ observed in the 50% O_2_ + 50% CO_2_ environment suggests enhanced passive film stability, as evidenced by lower passivation current density (*i*_p_).

The pitting potential (*E*_b_) is a critical parameter marking the threshold at which the metastable pitting transitions to stable pitting in stainless steel^30^. Repassivation potential (*E*_p_) defines a critical threshold for pitting growth^31^. Below *E*_p_, pitting is unlikely to occur, whereas above it, metastable pitting can initiate, and existing stable pitting may propagate. A larger difference (*∆E*) between *E*_b_ and *E*_p_ indicates greater susceptibility to passive film damage and reduced self-repair capacity^32^. In the 50% O_2_ + 50% CO_2_ environment, Super 13Cr stainless steel exhibits higher *E*_b_ and lower *E*_p_, leading to a larger *∆E*. This suggests that passive film is chemically more stable in the O_2_ + CO_2_ environment, while once stable pitting initiates, repassivation becomes more challenging.

**Table 2:** Key parameters obtained in Fig. 1.

Environment	E_corr_ (V)	i_*p*_ (A‧cm^−2^)	E_b_ (V)	E_*p*_ (V)	∆E = E_b_- E_*p*_ (V)
50%N_2_ + 50%CO_2_	−0.466	5.32 × 10^−6^	0.235	0.107	0.128
50%O_2_ + 50%CO_2_	−0.215	3.06 × 10^−6^	0.253	0.901	0.163

### Potentiostatic polarization curves

**Fig. 2:**
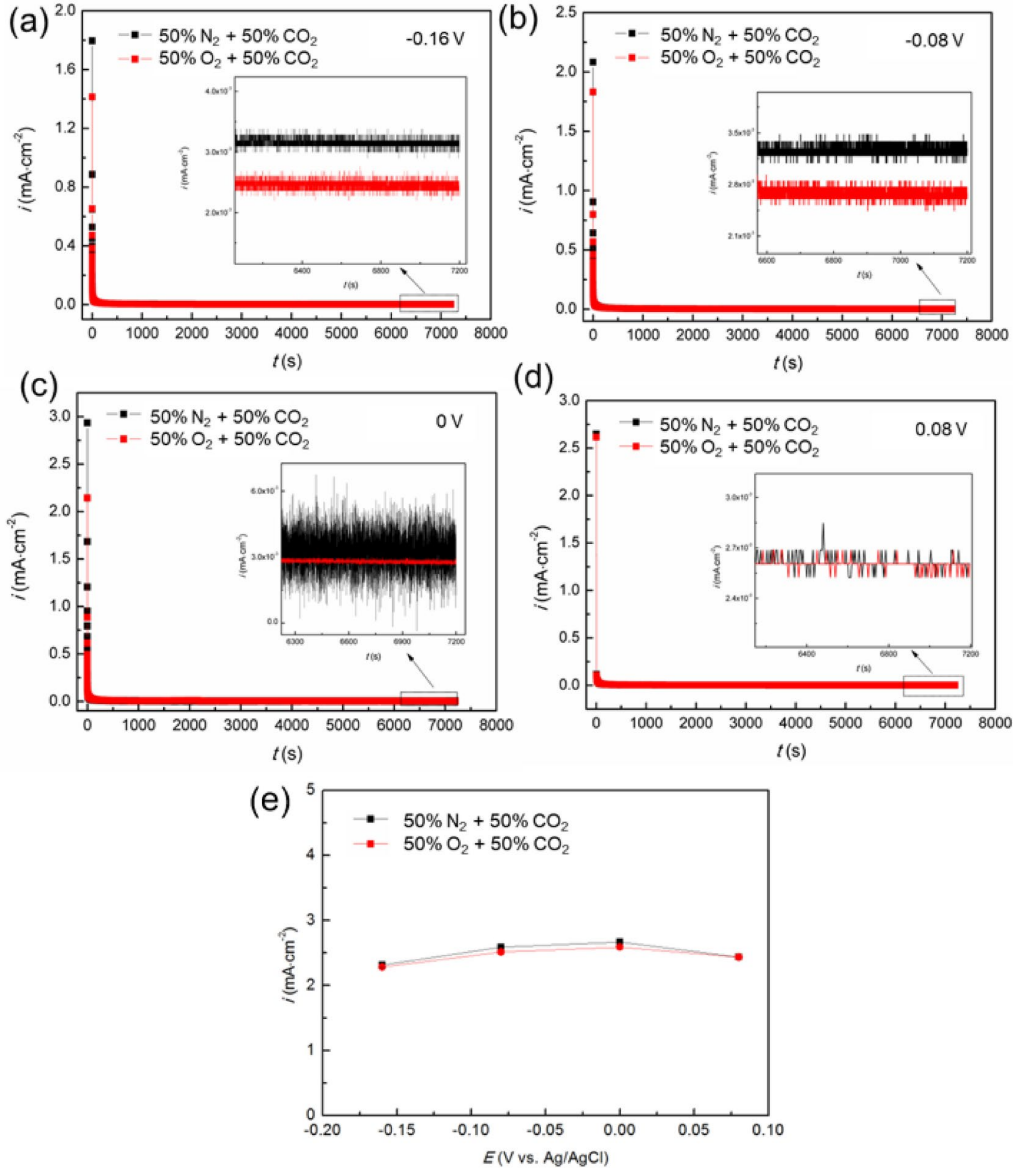
Potentiostatic polarization curves of Super 13Cr at various *E*_f_: (**a**) −0.16 V, (**b**) −0.08 V, (**c**) 0 V, and (**d**) 0.08 V for 2 h; (**e**) the correlation between *i*_ss_ and *E*_f_.

Based on the cyclic potentiodynamic polarization curve tests (Fig. 1), potentiostatic polarization was conducted at different *E*_f_ within the complete passivation region (*E*_corr_ to *E*_p_): −0.16, −0.08, 0 and 0.08 V. The tests were performed in a 50% O_2_/N_2_ + 50% CO_2_ environment for 2 h, as depicted in Fig. 2. As demonstrated in Fig. 2(a-d), the potentiostatic polarization curves exhibit a pronounced initial decrease in current density, which can be attributed to the rapid formation and growth of the passive film. Over time, the current density on all polarization curves tended toward stabilized as the rates of passive film formation and dissolution reached equilibrium. The absence of prominent current peaks in the curves indicates that metastable pitting did not occur at any applied *E*_f_ values for Super 13Cr.

Figure 2(e) displays the steady-state current densities (*i*_ss_) at various *E*_f_. For n-type passive film with metal interstitial ions and oxygen vacancies as the predominant point defects, the passivation current density is anticipated to be unaffected by the *E*_f_^33^. The data in Fig. 2(e) reveal that, regardless of the gaseous environment, the *i*_ss_ of Super 13Cr remains constant, which suggests that the passive films formed under both environments share the same n-type semiconductor characteristics.

### EIS tests

**Fig. 3:**
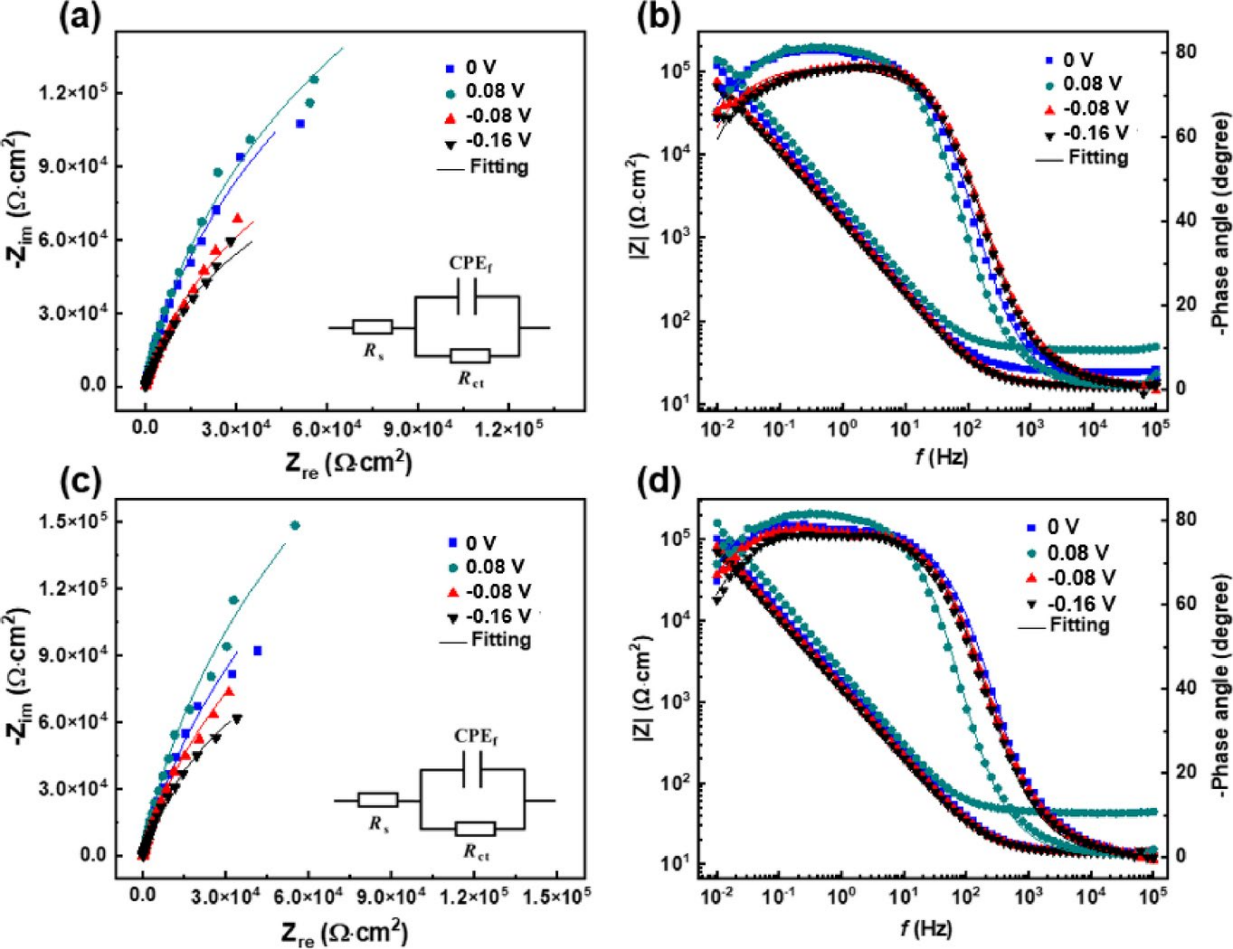
EIS results of Super 13Cr under various environments: (**a**, **b**) 50% N_2_ + 50% CO_2_, (**c**, **d**) 50% O_2_ + 50% CO_2_; (**a**, **c**) Nyquist plots and (**b**, **d**) Bode plots.

Figure 3 displays the EIS results of Super 13Cr stainless steel after potentiostatic polarization. The impedance arc radius for Super 13Cr increases with increasing *E*_f_, indicating enhanced protection. Impedance data were analyzed with the equivalent electrical circuit (EEC) inserted in the Nyquist plots (Fig. 3a, c)^34–36^, and summarized in Table 3. *R*_s_ and *R*_ct_ denote the solution and charge transfer resistance^37^, *CPE*_f_ corresponds to the passive film capacitance. At a given *E*_f_, the *R*_ct_ of Super 13Cr in the 50% O_2_ + 50% CO_2_ environment is higher than that in the 50% N_2_ + 50% CO_2_ environment, suggesting that oxygen significantly enhances passive film stability. The results align with the established understanding that passive film properties are pivotal in determining corrosion resistance of stainless steels, as supported by literature^33,38^.

**Table 3:** The fitted parameters in Fig. 3.

Conditions	E_f_ (V)	*R*_s_ (Ω·cm^2^)	*R*_ct_ (10^4^ Ω·cm^2^)	C_f_ (10^−5^ F·cm^−2^)	*n*	L_ss_ (nm)
50%N_2_ + 50%CO_2_	−0.16	13.23	17.53	1.73	0.86	0.61
	−0.08	13.38	22.92	1.60	0.86	0.69
	0	13.62	39.41	1.25	0.9	0.89
	0.08	13.52	49.40	0.83	0.91	1.59
50%O_2_ + 50%CO_2_	−0.16	13.44	24.58	1.51	0.86	0.87
	−0.08	13.49	41.97	1.37	0.86	0.96
	0	13.54	58.31	1.15	0.87	1.14
	0.08	13.67	67.52	0.78	0.91	1.69

The formula is used to deduce the relationship between the thickness of the passive film (*L*_ss_) and *CPE*_f_^39–41^:

1 $$\:{{L}}_{{ss}}{=}\frac{\varepsilon_{0}\:\varepsilon\:{{A}}}{{{CPE}}_{{f}}}$$

**Fig. 4:**
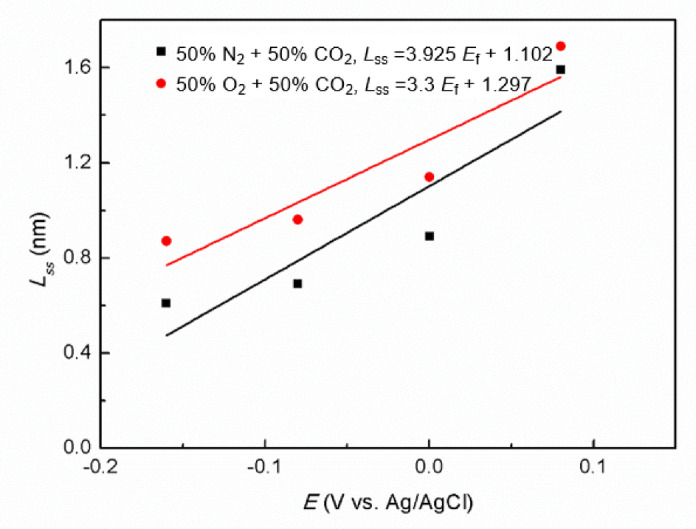
Relationship between *E*_f_ and *L*_ss_ of Super 13Cr in 0.1 M NaCl solution in N_2_/O_2_ + CO_2_ environment.

Here, *ε*_0_ and *ε* are the vacuum permittivity (8.854 × 10^−14^ F·cm^−1^) and dielectric constant (taken as 15.6^42^), and *A*(= 1 cm^2^) is exposed specimen area. The calculated values of *L*_ss_ are compiled in Table 3 and are consistent with the thickness ranges reported in the literature. Our findings reveal that, for a given *E*_f_, the *L*_ss_ in O_2_ + CO_2_ environment exceeds that formed in N_2_ + CO_2_ environment. The observation suggests that oxygen facilitates the development of the passivation, thereby enhancing passivation performance. The relationship between *L*_ss_ and *E*_f_ for Super 13Cr stainless steel was linear fitted (Fig. 4), and the correlation followed the equation^43^:

2 $$\:{{L}}_{{ss}}{=}\frac{{1}}{\varepsilon_{{L}}}(1-\alpha){{E}}_{{f}}{+B}$$

The average electric field strength (*ε*_L_) was determined using polarization rate ($\alpha$) at the passive film/solution interface, which is reported as 0.743^44^. The relationship is given by Eq. (2), where *B* is a constant specific to the system. The values of *ε*_L_ were calculated for the two different environments: 0.78 × 10^6^ V‧cm^−1^ for 50% O_2_ + 50% CO_2_ environment and 0.65 × 10^6^ V‧cm^−1^ for 50% N_2_ + 50% CO_2_ environment using this equation, suggesting that oxygen significantly enhances the *ε*_L_ within passive film on Super 13Cr stainless steel.

### M-S analysis

**Fig. 5:**
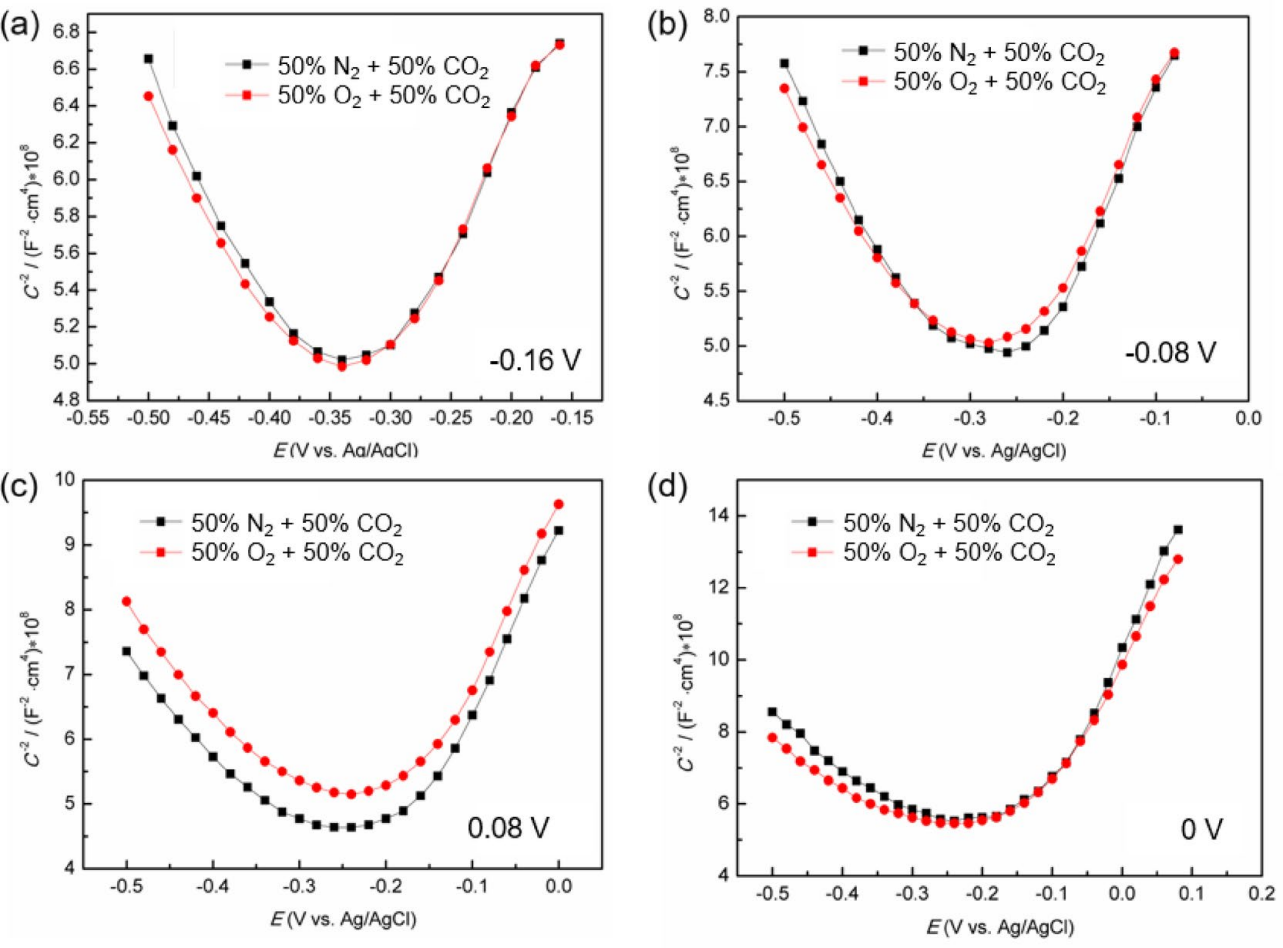
M-S curve of Super 13Cr in 0.1 M NaCl solution in N_2_/O_2_ + CO_2_ environment after potentiostatic polarized at (**a**) −0.16 V, (**b**) −0.8 V, (**c**) 0 V, and (**d**) 0.8 V for 2 h.

The PDM posits the semiconductor behavior of passive film. The susceptibility of the film to rupture, and consequently the initiation of pitting, is intrinsically connected with semiconductor properties. The space charge capacitance (*C*) and potential (*E*) are interrelated as follows:

3 $$\:\frac{{1}}{{{C}}^{{2}}}{=}\frac{{2}}{\varepsilon\varepsilon_{{0}}{e}{{N}}_{{D}}}\bigg({E}{-}{{E}}_{{f}}-\frac{{kT}}{{e}}\bigg) \qquad\qquad\qquad \text{n-type}$$

4 $$\:\frac{{1}}{{{C}}^{{2}}}=-\frac{{2}}{\varepsilon\varepsilon_{{0}}{e}{{N}}_{{A}}}\bigg({E}{-}{{E}}_{{f}}-\frac{{kT}}{{e}}\bigg) \qquad\qquad\qquad \text{p-type}$$

where *e* is the elementary charge (1.602 × 10^−19^ C), *N*_D_ and *N*_A_ represent the concentrations of donors and acceptors, respectively. *E*_fb_ is flat band potential, *k* denotes Boltzmann constant, and *T* represents absolute temperature. According to PDM, the passive film formed on stainless steel is considered as semiconductor oxide layer, filled with point defects that significantly influence the film’s integrity and its susceptibility to pitting corrosion. The model delineates the passive film into two distinct layers: inner barrier and outer precipitation layer. Inner barrier layer, predominantly composed of Cr_2_O_3_ with a high point defects density, is crucial for protective properties. For Super 13Cr stainless steel, the composition of barrier layer is characterized as Cr_2_-_*x*_Fe_*x*_O_3_, reflecting the higher Fe content relative to conventional austenitic stainless steels^45^. To maintain the stability of thickness and defect structure during M-S test, the reverse scan initiation potential was set to *E*_f_, with low scan rate. The region above − 0.4 V is selected to analysis the semiconductor properties of passive films to avoid alterations in the barrier layer properties at lower reverse scan potentials. Figure 5 presents the M-S results, demonstrating n-type under both experimental conditions, consistent with the potentiostatic polarization curve analysis (Fig. 2).

By linearly fitting the near-linear regions of the curves to extract the slopes, which facilitated the calculation of *N*_D_ and *N*_A_ via Eqs. (5) and (4). Across both the 50% O_2_ + 50% CO_2_ and 50% N_2_ + 50% CO_2_ environments, the higher *E*_f_ means the lower *N*_D_. The trend is indicative of a reduced density of point defects, which is attributed to the annealing effect of anodic polarization^46^, which is known to eliminate certain point defects. A decrease in *N*_D_ is beneficial as it mitigates the propensity for film rupture and pitting initiation. Thus, O_2_-enriched environment (50% O_2_ + 50% CO_2_) improves the passive film stability by diminishing point defects density, thereby reducing film rupture risk.

**Fig. 6:**
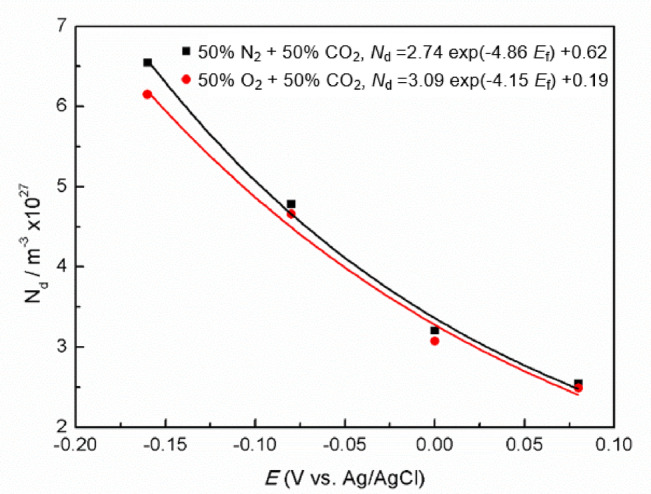
Relationship of *N*_D_ and *E*_f_ in 0.1 M NaCl solution of Super 13Cr in N_2_/O_2_ + CO_2_ environment.

The dependence of donor concentration (*N*_D_) on *E*_f_ was characterized using an exponential function^47^, as illustrated in Eq. (5). The parameters *ω*_1_, *ω*_2_, and *b* were determined through the exponential fitting of the *N*_D_-*E*_f_ curve, with the results depicted in Fig. 6.

5 $$\:{{N}}_{{D}}{=}{\omega}_{{1}}{exp}\left(-{b}{{E}}_{{f}}\right){+}{\omega}_{{2}}$$

The fitting outcomes are presented in Table 4. The relationship between *ω*_2_ and the diffusion coefficient (*D*_0_) of point defects is delineated by Eq. (6), which is pivotal for understanding the mobility of defects within the passive film^43^.

6 $$\:{{D}}_{{0}}=-\frac{{{J}}_{{0}}}{{2 K}\omega_{{2}}}=-\frac{{{J}}_{{0}}{RT}}{{2 F}{{\varepsilon}_{{L}}\omega}_{{2}}}$$

7 $$\:{{J}}_{{0}}=-\frac{{{i}}_{{ss}}}{{2}{e}}$$

The steady-state flux of point defects (*J*_0_) and the constants, including the gas constant (*R*) and the Faraday constant (*F*), are integral to the calculation of the *D*_0_, as defined by Eqs. (5) and (6). The *D*_0_ for the passive film on Super 13Cr under both 50% O_2_/N_2_ + 50% CO_2_ environments at various *E*_f_ could be determined utilizing the equations with the established parameters. Our findings align with the reported *D*_0_ values for the passive films on carbon steel in borate buffer solution as studied by Cheng et al.^48^, and for 316 L stainless steel in similar solutions, as researched by Feng et al.^49^, in terms of the order of magnitude. The consistency validates our experimental approach and supports the reliability of our results. In conclusion, as detailed in Table 4, O_2_ significantly influences passive film characteristics by reducing film rupture propensity and enhancing the overall corrosion resistance of passive film via reducing point defects density.

**Table 4:** Calculate parameters of super 13Cr under different *E*_f_ of 0.1 M NaCl in N_2_/O_2_ + CO_2_ environment.

Conditions	Parameters	E_f_ (V)	i_ss_ (10^−6^ A·cm^−2^)	D_0_ (10^−16^ cm^2^·s^−1^)
50%N_2_ + 50%CO_2_	*ω*_1_ = 2.74 *ω*_2_ = 0.62 *b*=−4.86	−0.16	2.475	2.46
		−0.08	2.628	2.61
		0	2.551	2.54
		0.08	2.398	2.38
50%O_2_ + 50%CO_2_	*ω*_1_ = 3.09 *ω*_2_ = 0.19 *b*=−4.15	−0.16	2.475	6.69
		−0.08	2.551	6.90
		0	2.628	7.10
		0.08	2.475	6.69

### Artificial pitting tests

**Fig. 7:**
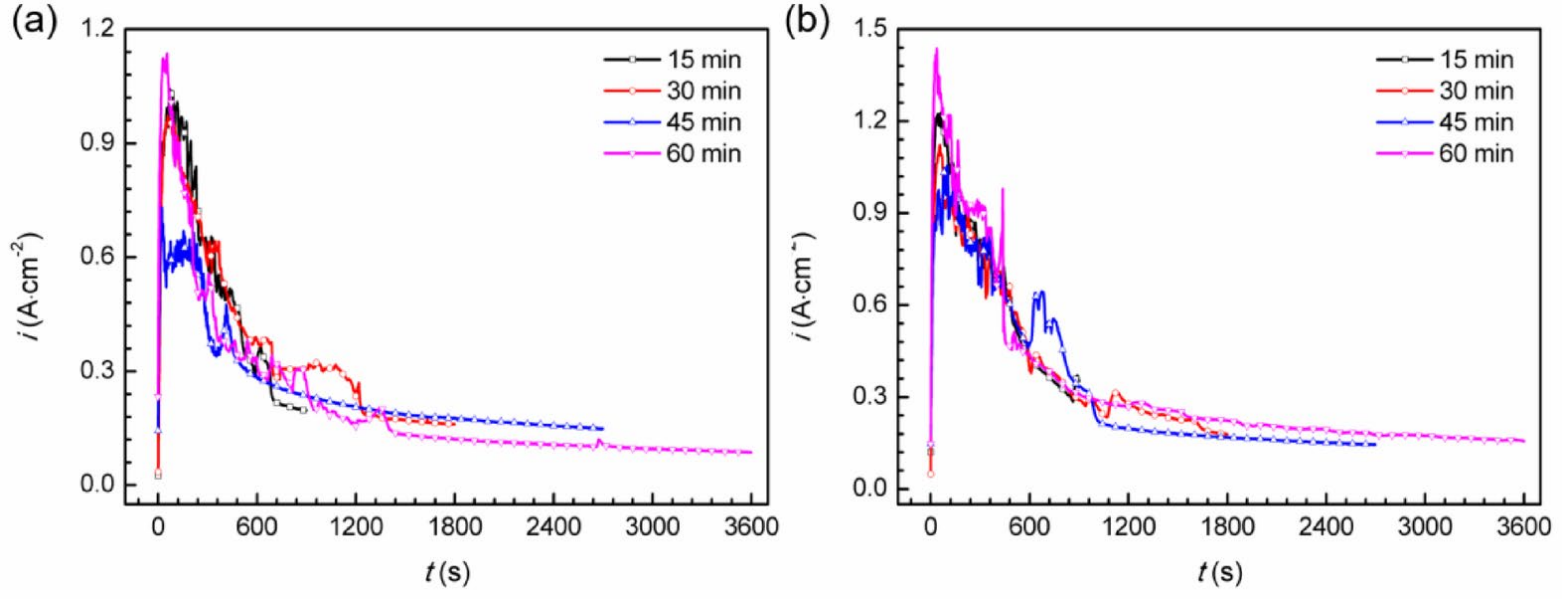
Potentiostatic polarization curves of Super 13Cr at 0.75 V, (**a**) N_2_ + CO_2_ (**b**) O_2_-CO_2_ environment.

An artificial pitting electrode was employed to potentiostatic anodically polarize the samples at potential of 0.75 V for varying durations (15, 30, 45 and 60 min), as depicted in Fig. 7. At the start of the polarization process, the current density surpassed 1 A·cm^−2^, indicating the initiation of stable pitting due to the high polarization potential applied. During polarization, the current density decreased and eventually reached a steady state. The observed trend is attributed to the progressive formation of FeCl_2_ salt layer at the base of the artificial pitting electrode. As the pit deepened, the rate-controlling step for stable pitting propagation shifted from activation-controlled to diffusion-controlled. The stabilization of the current density coincided with its approach to the limiting diffusion current density (*i*_L_) value.

**Fig. 8:**
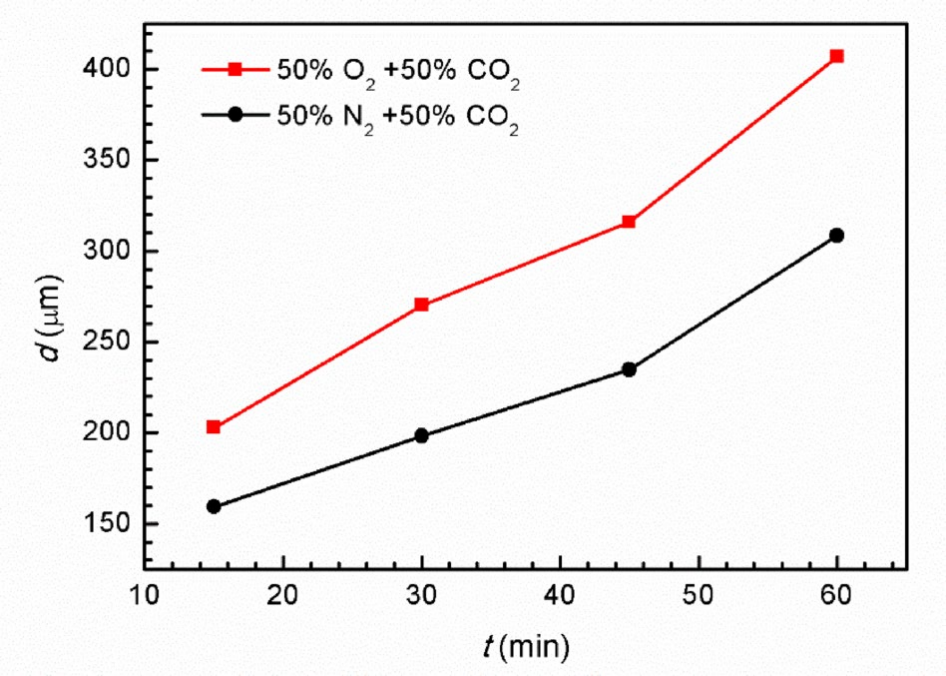
Pit depth (*d*) calculated based on Eq. (8).

Utilizing Faraday’s law, the depth of artificial pitting on Super 13Cr stainless steel was determined. The calculation is predicated on Eq. (8), wherein *Z*, representing the molar mass of Super 13Cr (55.8 g‧mol^−1^). *n* (= 2.16), corresponding to the average valence state of the cations. Additionally, *ρ*, the average density of Super 13Cr, is specified as 7.85 g‧cm^−3^. The outcomes of these calculations are graphically represented in Fig. 8.

8 $$\:{d=}\frac{{Z}}{{nF}\rho}\int\:{i}{dt}$$

Figure 8 demonstrates that the depth of artificial pitting on the Super 13Cr stainless steel electrode linearly increases with potentiostatic anodic polarization time, suggesting that O₂ enhances the anodic dissolution process during the steady-state pitting.

**Fig. 9:**
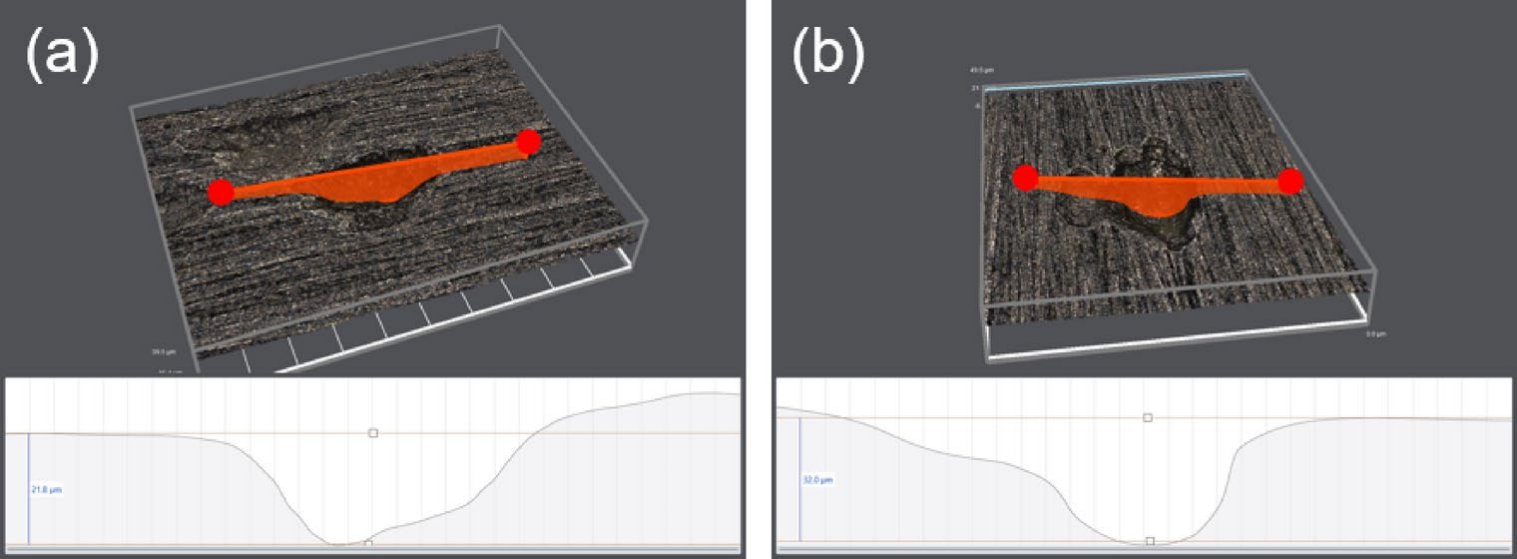
Super depth of field microscope images of Super 13Cr stainless steel in (**a**) N_2_ + CO_2_ and (**b**) O_2_ + CO_2_ environment.

Additionally, Fig. S1 presents the measured pitting depths on the artificial pitting electrode of Super 13Cr after various polarization durations. These measurements closely match the depths calculated using Faraday’s law, with discrepancies within an acceptable error margin of 15%. Figure 9 shows the super depth of field microscope images of Super 13Cr after constant-current anode polarization (1 mA/cm^2^) for 1 h in both environments. Large pits were observed in both environments, but the pit on Super 13Cr in the O_2_ + CO_2_ environment had a larger diameter and greater depth, consistent with the results obtained from Eq. (8). Moreover, the accumulation of corrosion products on the surface was not observed in either environment, as reported in the literature^50,51^, indicating that Cl⁻ inhibited the formation of FeCO_3_.

**Fig. 10:**
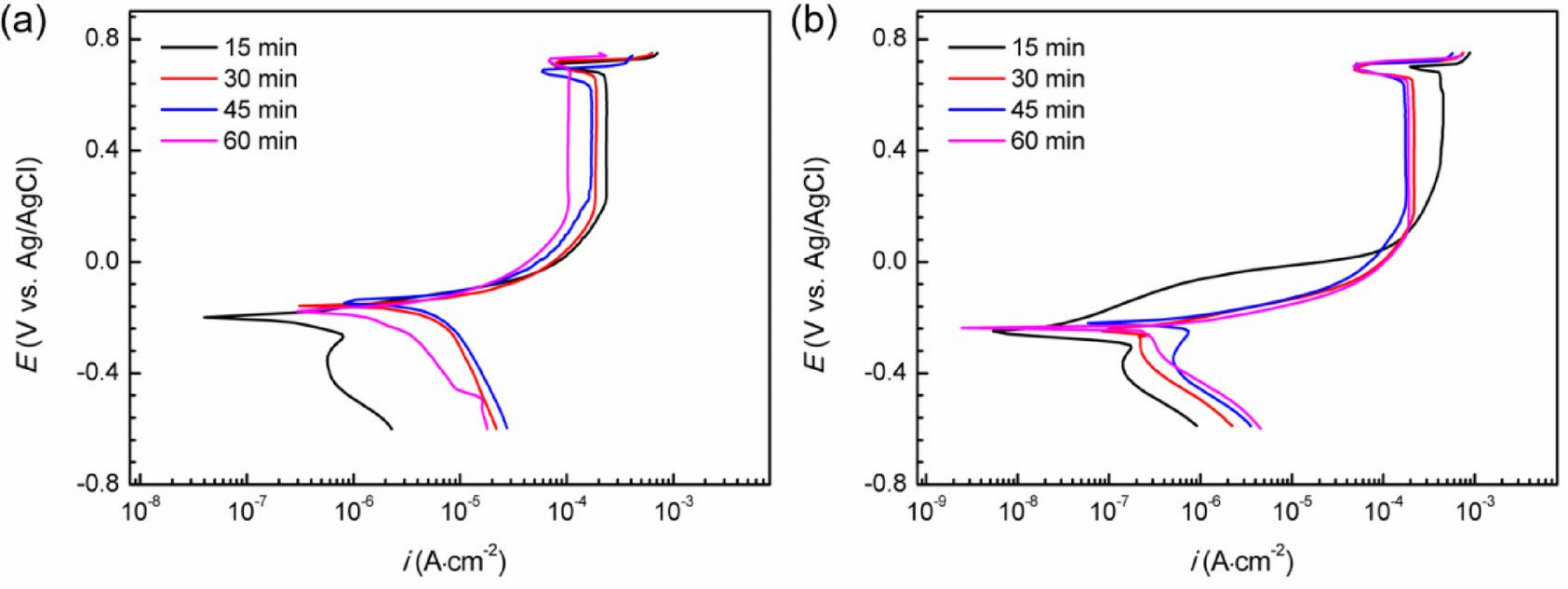
Cathodic back sweep curves of Super 13Cr after potentiostatic polarization at 0.75 V, (**a**) N_2_ + CO_2_; (**b**) O_2_ + CO_2_ environment.

Figure 10 presents the data of the cathodic scan subsequent to the establishment of steady-state pitting growth under potentiostatic anodic polarization at 0.75 V for varying durations. At the start of the scan, the Super 13Cr surface is covered with FeCl_2_ salt film, which controls the anodic dissolution rate through diffusion of Fe^2+^, maintaining a stable current density (*i*_L_). As the polarization potential progressively diminishes, the FeCl_2_ salt film on the Super 13Cr stainless steel dissolves, resulting in a significant drop in current density. The potential at which the transition occurs signifies the cessation of steady-state pitting, is defined as the repassivation potential (*E*_rp_).

**Fig. 11:**
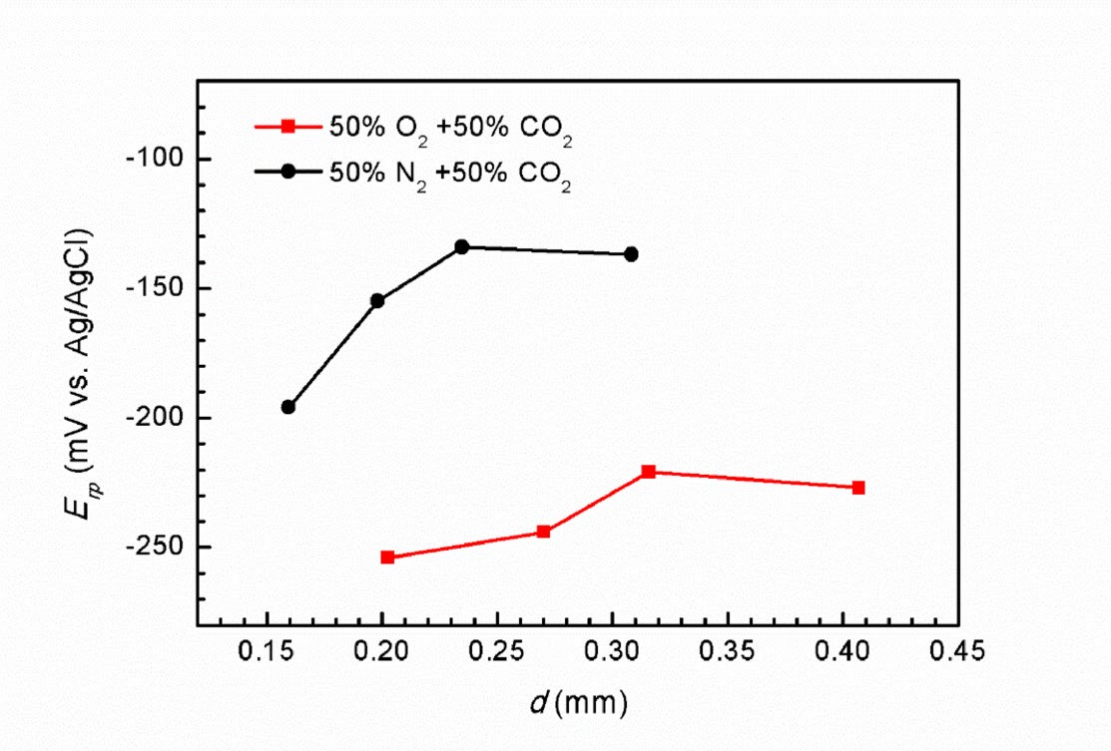
*E*_rp_ of Super 13Cr in N_2_/O_2_ + CO_2_ environment.

Figure 11 depicts the relationship between the *E*_rp_ and *d* for Super 13Cr stainless steel in two distinct environments. In each environment, *E*_rp_ initially increases with increasing pitting depth, followed by stabilization. The trend is associated with the steady-state pitting growth process, during which the anodic charge within the pits accumulates. Once a critical anodic charge is discharged, *E*_rp_ becomes independent of pitting depth, indicating a dynamic equilibrium between dissolution and repassivation^52^. In the 50% N_2_ + 50% CO_2_ environment, the *E*_rp_ of Super 13Cr stainless steel stabilizes at approximately − 0.13 V. Conversely, in the 50% O_2_ + 50% CO_2_ environment, the *E*_rp_ is notably lower, at about − 0.22 V. The reduction in *E*_rp_ in the presence of O_2_ suggests a reduced capacity for repassivation in Super 13Cr stainless steel after the onset of stable pitting. The observation is consistent with preliminary insights from the Fig. 1.

**Fig. 12:**
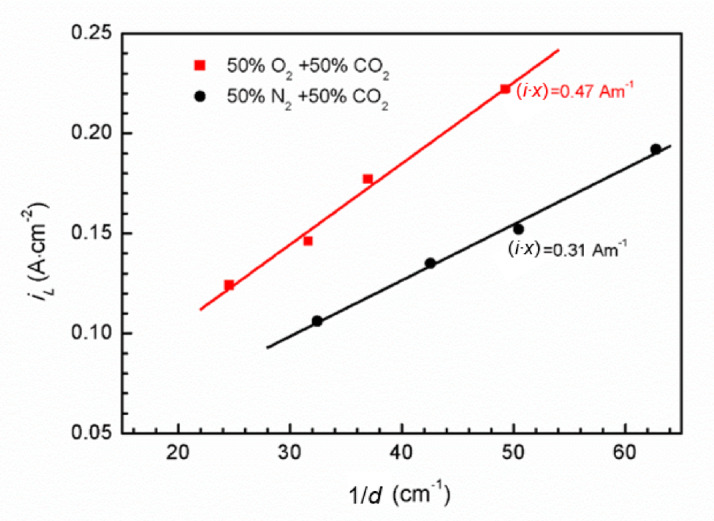
Relation between *i*_L_ and 1/*d* in 0.1 M NaCl solution of Super 13Cr in N_2_/O_2_ + CO_2_ environment.

The pitting stability product (*i*·*x*)_saltfilm_, which represents pitting corrosion stability under salt layer condition on metal surface, can be calculated using Eq. (9) as follows^53^.

9 $$\:{{i}}_{{L}}{d=nFD}\Delta {C}=({i}\cdot {x})_{{saltfilm}}$$

where *D* signifies the diffusion coefficient, and Δ*C* represents the concentration difference of metal cations between pit bottom and bulk solution. Figure 12 illustrates the relationship between the *i*_L_ and 1/*d* for Super 13Cr stainless steel. An inverse relationship is observed, with *i*_L_ decreasing as *d* increases, signifying that the anodic dissolution during the steady-state pitting process is predominantly governed by the diffusion. For environments with identical pitting depths, the higher *i*_L_ values in the 50% O_2_ + 50% CO_2_ environment suggests that O_2_ enhances the diffusion kinetics. The linear correlation between *i*_L_ and 1/*d* implies that the product *i*_L_⋅*d* is constant. Linear regression analysis of *i*_L_ versus 1/*d* yields products of 0.47 A·m^−1^ for 50% O_2_ + 50% CO_2_ environment and 0.31 A·m^−1^ for the 50% N_2_ + 50% CO_2_ environment. According to Eq. (9), the product *i*_L_⋅*d* is equivalent to the pitting stability product (*i*·*x*)_saltfilm_.

In accordance with Burstein’s pitting corrosion theory^54^, the pitting stability product *i*⋅*a* remains constant during the steady-state pitting phase, with the decisive threshold for the transition from metastable to stable pitting ranging from 0.3 to 0.6 A·m^−1^. Our experimental findings align with the theoretical framework. The increased pitting stability product (*i*·*x*)_saltfilm_ for Super 13Cr in 50% O_2_ + 50% CO_2_ condition indicates that O_2_ raises the threshold for the metastable pitting stability product *i*⋅*a* to reach the critical value necessary for the transition to stable pitting. The phenomenon also rationalizes the observation of higher pitting potential (*E*_b_) in the 50% O_2_ + 50% CO_2_ environment, as depicted in Fig. 1.

## Discussion

### O_2_ promotes passivation film densification

**Fig. 13:**
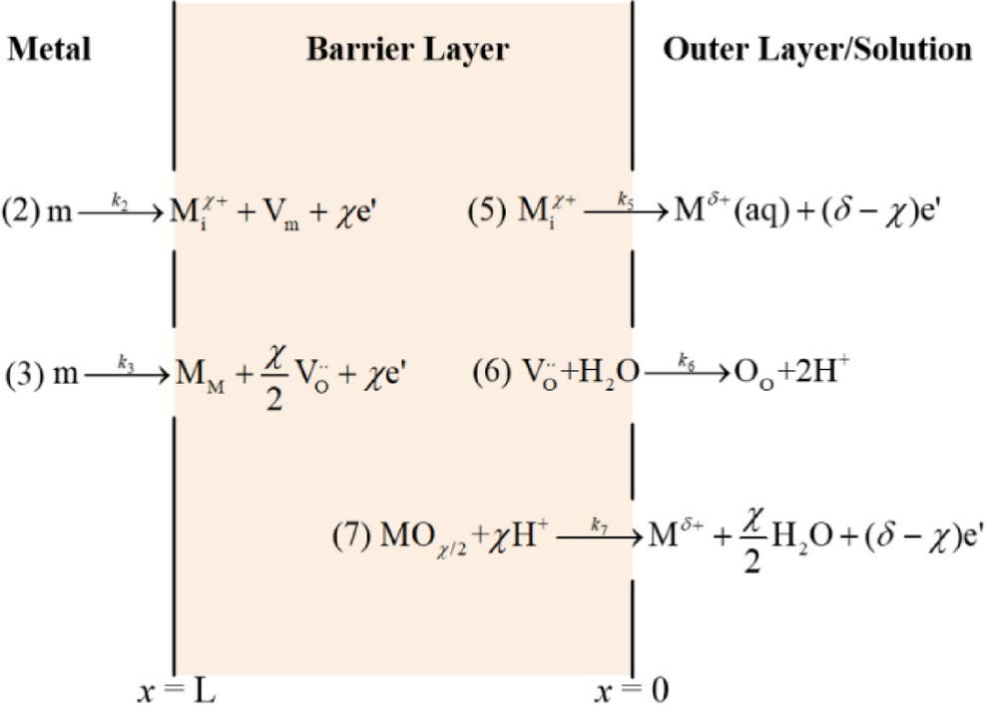
Reaction diagram of N-type semiconductor passivation film based on PDM.

Figure 13 illustrates the reaction mechanism of n-type passive film, as proposed by the PDM^33^. The diagram shows atomic-scale reactions at lattice points within the passive film’s crystal structure, particularly at matrix/barrier layer interface (m/bl) and the barrier/outer layer (solution) interface (bl/ol). In p-type semiconductor films, reactions (1) *M* + *V*_M_^*x*′^→*M*_M_+*V*_M_+*xe*′ and (4) *M*_M_ → *M*^*x*+^ +*V*_M_^*x*′^ characterize the behavior of metal vacancies $\:{\text{V}}_{\text{M}}^{x{\prime\:}}$. In contrast, for n-type semiconductors, the primary point defects are oxygen vacancies ($\:{\text{V}}_{\text{o}}^{}$) and metal interstitial ions ($\:{\text{M}}_{\text{i}}^{\text{x}\text{+}}$), rendering reactions (1) and (4) inapplicable. The reactions depicted in Fig. 13 are as follows:

Reaction (2) and Reaction (5) signify the generation of metal interstitial ions and the entry into bulk solution, respectively. Reaction (3) and (6) portray the creation of $\:{\text{V}}_{\text{o}}^{}$ at m/bl and the filling through the capture of oxygen ions from H_2_O at the bl/ol. Reaction (7) illustrates the dissolution process of the oxide film at the bl/ol, producing metal cations and H_2_O. Here, *m* denotes metal atoms, *V*_m_ represents metal atom vacancies, *M*^*δ*+^(aq) refers to metal cations in the solution, *M*_M_ indicates metal cations situated at the lattice points of the passive film, *O*_O_ signifies oxygen ions at the lattice points, and *MO*_χ/2_ represents stoichiometric oxide passive films.

In the N_2_/O_2_ + CO_2_ environment, the barrier layer on Super 13Cr exhibits characteristics of an n-type semiconductor, with predominant point defects being $\:{\text{V}}_{\text{o}}^{}$ and iron interstitial ions ($\:{\text{Fe}}_{\text{i}}^{\text{2+}}$). Given the larger ionic radius of Fe compared to O, $\:{\text{Fe}}_{\text{i}}^{\text{2+}}$ requires more activation energy to penetrate the passive film barrier layer than $\:{\text{V}}_{\text{o}}^{}$. The latter, acting as charge carriers, exhibit higher mobility within the passive film. Consequently, across both environments, $\:{\text{V}}_{\text{o}}^{}$ emerges as the dominant point defect. In O_2_ + CO_2_ condition, reaction (6) not only annihilates $\:{\text{V}}_{\text{o}}^{}$ at the bl/ol but also facilitates the adsorption of oxygen from solution into $\:{\text{V}}_{\text{o}}^{}$, following the reaction $\:{\text{V}}_{\text{o}}^{}$+O_2_→*O*_O_+O. The process accelerates the annihilation of $\:{\text{V}}_{\text{o}}^{}$. Additionally, O_2_ enhance *D*_0_ of point defects, enabling $\:{\text{V}}_{\text{o}}^{}$ to diffuse more readily from m/bl to bl/ol. Furthermore, at equivalent film-forming potentials, the generation rate of $\:{\text{V}}_{\text{o}}^{}$ at m/bl via the reaction Cr→Cr_Cr_+$\:\frac{\text{3}}{\text{2}}{\text{V}}_{\text{o}}^{}$+3$\:{e}^{{\prime\:}}$ remains consistent^55^, indicating that the rate of $\:{\text{V}}_{\text{o}}^{}$ generation is invariant regardless of O_2_ presence in the CO_2_ environment. Collectively, these findings suggest that in O_2_-enriched environments, $\:{\text{V}}_{\text{o}}^{}$ are more prone to diffusion and annihilation at the bl/ol, thereby enhancing passivation performance.

### O_2_ promotes the expansion of steady-state pitting

**Fig. 14:**
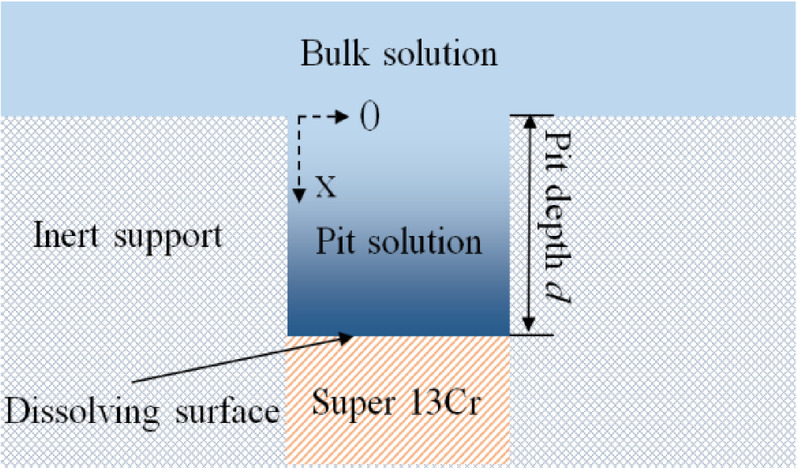
Geometry of artificial pit electrode based on Galvele local acidification model.

Once stable pitting corrosion is initiated in stainless steel, the corrosion process within the pit continues due to the creation of a localized, aggressive, and autocatalytic corrosive environment. A key parameter for assessing the intensity of the localized environment is the pH within the pit. Galvele’s local acidification model^56–58^ posits a mass transfer process limited to ionic diffusion, neglecting electromigration and convective effects, thereby simplifying the scenario to one-dimensional steady-state diffusion, as shown in Fig. 14. The hydrolysis of Fe^2+^, resulting from anodic metal dissolution, is assumed to proceed via a single step reaction. From the local acidification model, Eq. (10) is derived, which includes the following relationships: $\:{\text{D}}_{{\text{Fe}}^{\text{2+}}}$ represents the diffusion coefficient of Fe^2+^, $\:{\text{C}}_{{\text{Fe}}^{\text{2+}}}$ denotes the saturated solubility of FeCl_2_, quantified at 4.2 M, $\:{\text{D}}_{{\text{H}}^{\text{+}}}$ is the diffusion coefficient of H^+^, $\:{\text{C}}_{{\text{H}}^{\text{+}}}$ signifies the concentration of H^+^ at the base of pit, with local pH defined as the negative logarithm of $\:{\text{C}}_{{\text{H}}^{\text{+}}}$, *i*_L_‧d corresponds to the pitting stability product at the inception of stable pitting, *x*_1_ defines the boundary condition at *d* = 0, reflecting the product of the $\:{\text{C}}_{{\text{H}}^{\text{+}}}$ and $\:{\text{D}}_{{\text{H}}^{\text{+}}}$.

10 $${{D}}_{{{{Fe}}^{{{{2+ }}}}}} \cdot {{ C}}_{{{{Fe}}^{{{{2+ }}}} }} {{ + D}}_{{{{H}}^{{{+ }}} }} \cdot {{ C}}_{{{{H}}^{{{+ }}} }} {{ = }}\frac{{{{i}}_{{{L}}} {{d}}}}{{{{nF}}}}{{ + x}}_{{{1}}}$$

For Super 13Cr stainless steel featuring a FeCl_2_ salt film at the base of artificial pits, metal anodic dissolution occurs during the steady-state pitting. The resultant Fe^2+^ undergo one-dimensional steady-state diffusion within the pit channel, extends to a depth of *d*. The concentrations of relevant ions comply with the conditions defined by Eq. (9). Given that the pH values of the bulk solution are identical in both environments, the boundary condition *x*_1_ remains consistent. The pitting stability product *i*_L_‧*d* at the base of the artificial pits on Super 13Cr stainless steel is directly proportional to the $\:{\text{C}}_{{\text{H}}^{\text{+}}}$ at pit bottom and inversely proportional to local pH. It has been established that in the 50% O_2_ + 50% CO_2_ environment, the *i*_L_‧*d* value (0.47 A·m^−1^) on Super 13Cr is higher than that in the 50% N_2_ + 50% CO_2_ environment (0.31 A·m^−1^). Consequently, the pH at the base of the artificial pits on Super 13Cr in the O_2_ + CO_2_ environment is lower, indicating a more aggressive local corrosive environment. The finding suggests that O_2_ exacerbates the severity of the local environment within pits where stable pitting occurs on Super 13Cr stainless steel.

## Conclusion

In the study, techniques such as cyclic potentiodynamic polarization, potentiostatic polarization, EIS, M-S analysis, and artificial pitting electrodes were employed to investigate the passive film properties of Super 13Cr in 0.1 M NaCl solution under O_2_/N_2_ + CO_2_ environments at ambient temperature. The effects of O_2_ on the passive film properties, the kinetic characteristics of steady-state pitting-repassivation behavior, and the underlying mechanisms were elucidated using the point defect model (PDM) and Galvele’s local acidification theory. The findings reveal the mechanisms governing passivation performance and pitting growth kinetics, as summarized below:

1. Upon achieving a fully passivated state, O_2_ promotes the development of the passive film, increasing its thickness, enhancing the *D*_0_ and reduces the *N*_D_, this diminishes the probability of film rupture and pitting initiation, thereby enhancing passivation performance.
2. During the transition process of metastable to stable pitting, O_2_ lowers the metastable pitting stability product (*i*·*a*) and raises the threshold for the transition. Specifically, the pitting stability product in the presence of a salt film, (*i*·*x*)_saltfim_, is increased. These dual effects inhibit the transformation to stable pitting.
3. In the stable pitting process, O_2_ reduces the repassivation potential and increases both the *i*_L_ and (*i*·*x*)_saltfim_, leading to a decreased pH level at the base of the pit, creating a more aggressive local corrosive environment. Consequently, this results in greater pit depth and an accelerated pitting growth rate, which in turn weakens the repassivation capability of Super 13Cr.

## Electronic supplementary material

Below is the link to the electronic supplementary material.

Supplementary Material 1

## Data Availability

The datasets used and/or analysed during the current study available from the corresponding author on reasonable request.
