# Supplementary figures and images for: Double effects of O2 on passive film of super 13Cr stainless steel in CO2 saturated environment
Source: Sci Rep. 2025 Jun 6;15:19870. doi: 10.1038/s41598-025-01208-7 (PMC12141685; doi:10.1038/s41598-025-01208-7)

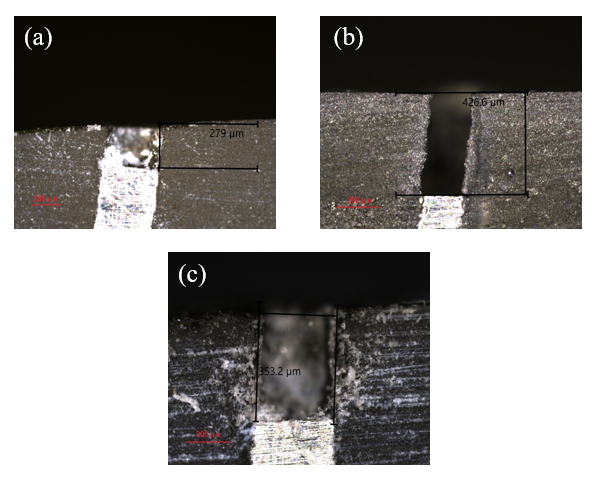


Fig.S1 Super 13Cr artificial pitting pit morphology in (a) 50%O2+50%CO2, 30min; (b) 50%O2+50%CO2, 60min; (c) 50%N2+50%CO2, 60min.

Supplement: Supplementary file 1 — Supplementary Material 1 [file 41598_2025_1208_MOESM1_ESM.docx]
